# Occurrence and identification of hemotropic mycoplasmas (Hemoplasmas) in free ranging and laboratory rats (*Rattus norvegicus*) from two Brazilian zoos

**DOI:** 10.1186/s12917-015-0601-8

**Published:** 2015-11-23

**Authors:** Francisco de Oliveira Conrado, Naíla Cannes do Nascimento, Andrea Pires dos Santos, Cristina Kraemer Zimpel, Joanne Belle Messick, Alexander Welker Biondo

**Affiliations:** Programa de Pós-Graduação em Biologia Celular e Molecular, Universidade Federal do Paraná, Avenida Coronel Francisco H dos Santos, Jardim das Américas, Curitiba, Paraná CEP 81531-900 Brazil; Departamento de Medicina Veterinária, Laboratório de Zoonoses e Epidemiologia Molecular, Universidade Federal do Paraná, Rua dos Funcionários 1540, Bairro Juvevê, Curitiba, Paraná CEP 80035-050 Brazil; Hemoplasma Laboratory, Purdue University, College of Veterinary Medicine, Department of Comparative Pathobiology, VPRB, Room 109, 725 Harrison Street, West Lafayette, IN 47905 USA; Director of Animal Services, Curitiba Secretary of Environment, Passeio Público, Rua Luíz Leão S/N, Centro, Curitiba, Paraná CEP 80030-010 Brazil

**Keywords:** Rodents, Hemoparasites, Mycoplasmal disease

## Abstract

**Background:**

Hemotropic mycoplasmas (hemoplasmas), bacteria belonging to the class Mollicutes, are obligatory red blood cell pathogens of a variety of animal species. They may cause acute anemia that is life-threatening or chronic disease that is clinically silent, but may interfere with results of experimental studies when using infected animals. Since these bacteria cannot be cultivated, molecular techniques are the gold standard for diagnosing an infection, investigating its prevalence, and describing new species. *Mycoplasma coccoides* and *M. haemomuris* are the most commonly recognized hemoplasmas in the blood of wild and laboratory rodents. Neither the epidemiology nor clinical and molecular characterization of hemoplasma infection in free-ranging rodents in Brazil has been previously reported. The aims of this study were to investigate the occurrence of hemoplasmas in free-ranging rats (*Rattus norvegicus*) captured in the Passeio Público and Curitiba Zoo and compare hematologic parameters of infected and non-infected animals.

**Results:**

Anti-coagulated blood samples collected from 43 free-ranging and 20 nursery rats were included in the study. Overall 63.5 % were positive using SYBR® Green quantitative PCR (qPCR) of the 16S rRNA gene to screen for hemoplasma infection (72 % among free-ranging rats; 45 % among laboratory-raised rats). Sequencing of the qPCR products showed that all but one sample had >98 % identity to *M. haemomuris*. Phylogenetic analysis based on a fragment of approximately 1300 bp of the 16S rRNA gene showed 99 % identity to a new hemoplasma from European rats and 98 % identity to a hemotropic mycoplasma described infecting a European harvest mouse (*Micromys minutus*). No statistically significant changes in hematologic parameters between infected and non-infected rats were found, confirming the low pathogenicity and/or silent characteristics of the infection.

**Conclusions:**

Our findings suggest that hemoplasmas are likely endemic in rodent species in this region. The epidemiology, especially as it relates to the mode of transmission, needs to be further investigated as well as the possibility that other animal species, including humans, might become infected.

## Background

Hemotropic mycoplasmas, also known as hemoplasmas, are the causative agents of infectious anemia in many mammalian species worldwide [1]. Previously divided in two genera (*Haemobartonella* and *Eperythrozoon*) in the Anaplasmataceae family, they were reclassified into the genus *Mycoplasma* based on 16S rRNA gene sequence data [2]. They are small, pleomorphic uncultivable bacteria that lack a cell wall and attach to the surface of red blood cells [3]. It is speculated that hemoplasmas cause disease by a nutrient scavenger and competition mechanism, which may lead to decreased life span of erythrocytes and severe anemia in acutely infected animals [4]. Hemoplasmas also can be opportunistic agents, silently infecting healthy animals and leading to disease only in certain conditions [3]. Natural transmission is believed to occur through blood-sucking arthropod vectors such as ticks, fleas, lice, mosquitoes and flies [2].

Several new species of hemoplasmas infecting wild and domestic animals were recently described [5–13]. Although most hemotropic mycoplasmas are host specific, interspecies infections [14] and zoonotic potential have been reported [15–17], supporting the importance of studying these microorganisms. Those individuals who are immunocompromised or have high exposure to arthropod vectors are at risk of being infected. A possible association between chronic hemoplasma infection and autoimmune diseases such as systemic lupus erythematosus, rheumatoid arthritis and rheumatic diseases in humans was reported in literature before the use of polymerase chain reaction (PCR). However, more recent cases of hemoplasma infection have been described using molecular techniques, these include *M. haemofelis*-like infection of a HIV-positive patient [15], *M. ovis* infection of an immunocompromised veterinarian with multiple sclerosis [18], *M. suis* infection of swine-farm workers in China [19], and infection of a patient with hemolytic anemia and pyrexia with a novel hemoplasma [17].

*Mycoplasma coccoides* (*Eperythrozoon coccoides*) and *M. haemomuris* (*Haemobartonella muris*) are red cell parasites of wild and laboratory rodents [2, 20–22]. A novel species of hemotropic mycoplasma was recently described in sewer rats (*Rattus norvegicus*) captured during rodent control in an animal hospital in Japan [12]. However, the prevalence of hemoplasma infections in free-ranging rodents remains largely unreported. Rodents, whether free-ranging or captive, may pose a risk to the health of human and other animal populations, as they can transmit a number of infectious diseases [23, 24]. The occurrence of hemoplasmas in free-ranging rodents in places where wild animals are kept in captivity, such as the Passeio Público and the Curitiba Zoo, might represent an important risk of transmission to workers and visitors as well as to animal species at the parks. Neither Passeio Público nor the Curitiba Zoo have efficient pest and rodent control plans. The keeping of animals in captivity at these sites hinders the use of toxic rodenticides or anticoagulants due to the risk of intoxication. The capture of live animals has been used as a method of palliative control of rodent infestation in these locations in an attempt to reduce the number of these animals.

Due to our inability to culture hemoplasmas *in vitro*, PCR is considered the gold-standard method for detection of infection. Microscopic observation of blood smears is also used, despite the low sensitivity of this technique [16]. Several protocols have been described for the diagnosis of hemotropic mycoplasma infection, including conventional PCR (cPCR) and quantitative PCR (qPCR) (SYBR® Green or TaqMan®). These techniques may be followed by Sanger sequencing to differentiate the infecting species [25, 26].

Many of the Brazilian domestic and wild animal species can be infected with hemoplasmas [27]. Additional studies are needed to help to establish mode(s) of transmission, host species or reservoirs of infection and its effects, as well as to identify new hemoplasma species. The aim of this study was to investigate the occurrence of hemotropic mycoplasmas in rats (*Rattus norvegicus*) captured in two parks in Curitiba and in laboratory rats by SYBR® Green qPCR. In addition, hematologic parameters, body weight, and location of infected rats were compared to those of non-infected animals.

## Methods

### Animals and blood collection

A total of 43 free-ranging rats (*Rattus norvegicus*) and 20 nursery rats were sampled from the city of Curitiba (Parana State, Brazil) for this study. The previously published hemoplasma infection percentage of 12.5 % in a population of free-ranging rats [12] was used to determine a minimum of 40 animals (95 % confidence level, with a 10 % interval) needed in this study. All samples were collected between the months of July 2013 and January 2014. Twenty-three rats were captured at the Passeio Público [10 (43 %) male and 13 (56 %) female], and 20 captured at the Curitiba Zoo [11 (55 %) male and 9 (45 %) female]. Rodent control (chemical and trapping) was responsibility of the Department of Animal Services, Curitiba Secretary of Environment, so no additional permission was necessary. In addition, 20 rats [12 (60 %) male and 8 (40 %) female] from the nursery kept at the Passeio Público for feeding the carnivores and originally from a laboratory rat supplier were sampled. The animals were captured using live traps that were set out overnight and collected in the morning; fruits and raw corn were used as baits. Upon capture, the animals were handled humanely and personal protection measures cautiously taken. The traps were put inside a hermetically closed plastic container and inhalation anesthetic Isoflurane infused through a machine with oxygen. The rats were considered sedated when all movement ceased and they became unresponsive to external stimuli. Once sedated, the animals were removed from the trap and kept sedated through a small inhalation mask. Blood was collected by intracardiac venipuncture into EDTA K2 and dry vacuum tubes (BD Brasil, São Paulo, Brazil) for hematologic analyses, DNA extraction and serum separation. After the blood collection the animals were euthanized with a lethal dose of intracardiac potassium chloride. During sampling, information concerning gender, weight (as an estimate of age) and place of capture were collected. Capture and use of animals as well as handling protocols were previously approved by the Ethics Committee on Use of Animals of the Federal University of Paraná under protocol number CEUA/SCA 057/2013.

### Microscopy

Blood smears were prepared from EDTA K2 anticoagulated blood within five minutes after blood collection to ensure no hemoplasma detachment from red blood cell surface [28]. The slides were stained using Romanowsky staining (May-Grünwald - Giemsa) in an automated stainer (Sysmex XE-2100, Sysmex Corporation, Japan) to ensure constant staining quality. Blood smear preparations were scanned for the presence of hemoplasmas using a motorized Axio Imager Z2 microscope (Carl Zeiss, Jena, DE) equipped with an automated scanning VSlide system (Metasystems, Altlussheim, DE) at the Multiuser Conventional and Confocal Fluorescence Microscopy Laboratory at the Department of Biological Sciences, Federal University of Paraná State, Brazil. For each slide, 1000 red blood cells were counted in different randomly chosen high power (100x) fields and the number of structures morphologically compatible with hemotropic mycoplasmas noted. Microscopy results for detection of hemoplasmas were subsequently compared with molecular PCR results.

### Hematologic analyses

Samples were stored at 4 °C until processed. Packed Cell Volume (PCV) was measured by routine centrifugation and Total Plasma Protein (TPP) was measured by refractometry.

### DNA extraction

Aliquots of whole blood anticoagulated with EDTA K2 were stored at −80 °C until the molecular procedures were performed. DNA extraction was performed at the Zoonoses and Molecular Epidemiology Laboratory, Department of Veterinary Medicine, College of Veterinary Medicine, Federal University of Paraná, Curitiba, Paraná, Brazil. DNA was extracted in duplicates from 100 μL whole blood using commercial Quick-gDNA™ MiniPrep Kit (Zymo Research Corp., Orange, California, USA) according to manufacturer’s instructions. For each 10 extractions of blood samples a negative control (ultrapure water) was included to ensure that there was no cross contamination. Blood sample from a known hemoplasma non-infected mouse was also extracted as negative control. The extracted duplicates of DNA were stored at −80 °C until they were sent to the Hemoplasma Laboratory of the Comparative Pathobiology Department, College of Veterinary Medicine at Purdue University, USA. Sample selection among duplicates was performed based on NanoDrop (Nd-1000 Spectrophotometer, Thermo Scientific, USA) nucleic acid concentration and purity results.

### GAPDH-PCR assay

A cPCR for the housekeeping gene glyceraldehyde-3-phosphate dehydrogenase (GAPDH) was performed to ensure successful DNA extraction and the presence of amplifiable DNA in the samples. Previously described primers [29] were aligned with the complete *Rattus norvegicus* genome using BLASTn tool to confirm identity to the murine GAPDH gene. A cPCR was carried out consisting of 15.875 μL of nuclease-free water, 2.5 μL of 10X Standard Taq Reaction buffer (1X), 0.5 μL of dNTPs (200 μM), 0.125 μL of Taq DNA Polymerase (1.25 units) (M0273S, NewEngland BioLabs® Inc., Ipswich, MA, EUA), 0.5 μL (0.2 μM) of forward primer, 0.5 μL (0.2 μM) of reverse primer and 5 μL of sample DNA. Cycling conditions were as follows: 95 °C for 30", 34 cycles of amplification (95 °C for 30 s, 55 °C for 30 s and 68 °C for 30 s), 68 °C for 5 min followed by cooling at 4 °C (iCycler® Thermal Cycler, Bio-Rad Laboratories Inc., Life Science Group, EUA).

### Gel electrophoresis and visualization

PCR products and a molecular weight marker (100 bp DNA Ladder Plus, Fermentas, Glen Burnie, Maryland, USA) were loaded into a 1.5 % agarose gel and separated by horizontal electrophoresis in 1X TAE buffer at 100 V and 3 W for one hour. Gels were stained in 0.5 μg/mL ethidium bromide solution under gentle agitation for 30 min. Gels were exposed to 312 nm UV light in a transilluminator for visualization of the separate fragments of the PCR product and size comparison with the molecular weight marker. Gels were photographed using the imaging system G:BOX Chemi XR5 (GBCXR50313, SYNGENE^©^, Frederick, MD, USA).

### Universal hemoplasma screening

A previously described SYBR® Green universal hemoplasma qPCR [30] was used to screen the extracted DNA samples for infection. The reaction was carried out in a qPCR machine (Applied Biosystems® 7300 Real-Time PCR System, Foster City, CA, EUA) according to Willi et al. [30] protocol. Ultrapure water and DNA extracted from known non-infected mouse were used as negative controls. Known positive samples for *Mycoplasma haemomuris* (kindly provided by Dr. Rikihisa, The Ohio State University) and for a hemotropic mycoplasma identified in capybaras (*Hydrochaeris hydrochaeris*) [5], were used as positive controls.

Each sample was analyzed in duplicate and the results considered positive when both crossed the threshold. Using the same pair of primers Willi et al. [30] reported nonspecific amplification products between cycle threshold (C_T_) 34.4 and 39.9 for the technique described. Thus, samples with C_T_s above 34 were reamplified by cPCR for confirmation. The reaction consisted of 15.875 μl of nuclease-free water, 2.5 μl 10X buffer, 0.5 μl dNTPs (200 μM), 0.125 μl Taq DNA Polymerase (1.25 units) (M0273S, NewEngland BioLabs® Inc. Ipswich, MA, USA), 0.5 μl forward primer (0.2 μM) (SYBR_For), 0.5 μl reverse primer [(0.2 μM) SYBR_Rev1 and SYBR_Rev2 in a 1:1 mix], and 5 μl of sample DNA, in a final volume of 25 μl. The protocol reaction was performed in a thermocycler (iCycler® Thermal Cycler, Bio-Rad Laboratories, Inc., Life Sciences Group, USA) and consisted of 30 s of denaturation at 95 °C followed by 40 amplification cycles (30 s at 95 °C, 30 s at 56 °C, and 30 s at 68 °C), and a final extension cycle of 5 min at 68 °C followed by cooling at 4 °C. Amplified products were subjected to electrophoresis and visualization as previously described.

### Sequencing

Twenty-six samples positive for hemoplasma by SYBR® Green qPCR underwent cPCR for amplification of a longer fragment and subsequent sequencing for comparison to 16S rRNA gene sequences available in GenBank®. In order to design primers for a rodent hemoplasma PCR assay, the 16S rRNA genes of the following hemotropic mycoplasma species were retrieved from GenBank® and aligned using the Genomatix^©^ software (Genomatix Software v. 3.1 GmbH 1998–2014): *M. haemomuris* (accession number U82963), *M. coccoides* (ac. number AY171918), Mycoplasma sp. N008 (sewer brown rats hemoplasma) (ac. number AB752303), ‘*Candidatus* M. haemomeles’ (Japanese badger hemoplasma) (ac. number AB848713), uncultured Mycoplasma sp. (Brazilian capybara #01 hemoplasma) (ac. numbers FJ667773 and FJ667774). Conserved regions among the sequences were identified and selected for primer design flanking a region of approximately 600 base pairs (bp). Primers RodHem F1 and RodHem R1 were designed using Primer3 software (http://primer3.ut.ee/) and analyzed by IDT’s Oligo Analyzer Tool (https://www.idtdna.com/calc/analyzer) and PCR Primer Stats (http://bioinformatics.org/sms2/pcr_primer_stats.html) software. All primers used in this study were commercially synthesized by IDT® (Integrated DNA Technologies, Coralville, IA, USA) and are described on Table 1. The cPCR reaction consisted of 45 μl of PCR SuperMix (PCR SuperMix High Fidelity, Invitrogen™, Life Technologies, USA), 1 μl (0.2 μM) of forward primer (RodHem F1), 1 μl (0.2 μM) of reverse primer (RodHem R1) and 5 of μL of DNA sample in a final volume of 52 μl/reaction. Cycling conditions consisted of 30 s at 94 °C followed by 35 amplification cycles (30 s at 94 °C, 30 s at 56 °C and 30 s at 72 °C) and a final extension step at 72 °C for 5 min, followed by cooling at 4 °C (PTC-200 Peltier Thermal Cycler, MJ Research, Inc., Waltham Massachusetts, USA). A positive sample for *M. haemomuris* was used as a positive control. A SYBR® Green negative sample and ultrapure water were used as negative controls. Amplified products were subjected to electrophoresis and visualization as previously described.

**Table 1:** Primers used in this study. Name, sequence and reference of primers used and/or designed and synthesized for use in this study

Primer	Sequence (5’-3’)	Reference
GAPDH-F	CCTTCATTGACCTCAACTACAT	Birkenheuer et al., 2003 [29].
GAPDH-R	CCAAAGTTGTCATGGATGACC	Birkenheuer et al., 2003 [29].
SYBR_For	AGCAATRCCATGTGAACGATGAA	Willi et al., 2009 [30].
SYBR_Rev1	TGGCACATAGTTTGCTGTCACTT	Willi et al., 2009 [30].
SYBR_Rev2	GCTGGCACATAGTTAGCTGTCACT	Willi et al., 2009 [30].
RodHem F1	GGGATTGAGATACGGCCCAT	This study.
RodHem R1	AGGTCCCCGTCAATTCCTTT	This study.
RodHem1300 Fw1	GCGAACGGGTGAGTAATGAA	This study.
RodHem1300 Fw2	GCAAACGGGCGAGTAATACA	This study.
RodHem1300 Rv	TCATAGTTTGACGGGCGGT	This study.

Approximately 10 % of the samples were also subjected to amplification of the nearly complete 16S rRNA gene sequence. Following alignment of 16S rRNA gene sequences of rodent hemotropic mycoplasmas as previously described, two conserved regions more distant from each other were selected in order to amplify a product with approximately 1300 bp for analysis and construction of a phylogenetic tree.

Primers RodHem1300 Fw1, RodHem1300 Fw2 and RodHem1300 Rv were designed and commercially synthesized as previously described. Two forward primers were used to assure amplification of different rodent hemoplasma rRNA 16S gene sequences despite nucleotide mismatches in this region. The cPCR reaction consisted of 45 μl of PCR SuperMix (PCR SuperMix High Fidelity, Invitrogen™, USA), 1 μl (0.2 μM) of forward primers (RodHem1300 Fw1 and RodHem1300 Fw2 in a 10 μM 1:1 mix) 1 μl (0.2 μM) of reverse primer (RodHem1300Rv) and 5 μl of sample DNA, totaling a 52 μl reaction. Cycling conditions consisted of 30 s at 94 °C followed by 35 amplification cycles (30 s at 94 °C, 30 s at 55.7 °C and 90 s at 72 °C) and a final extension step at 72 °C for 5 min followed by cooling at 4 °C (iCycler® Thermal Cycler, Bio-Rad Laboratories, Inc., Life Sciences Group, USA). Known positive and negative controls were included in the assay and products were separated and visualized as previously described.

Conventional PCR products visualized on the agarose gel were purified using the QIAquick Gel Extraction Kit (QIAGEN Inc., CA, USA) according to the manufacturer’s specifications and submitted for direct Sanger sequencing using the capillary DNA analyzer ABI 3730XL (Applied Biosystems, FosterCity, CA, USA) after sequencing reactions with a BigDye Terminator V3.1 cycle sequencing kit (Applied Biosystems). Reaction cleanup was performed via ethanol precipitation. Forward and reverse nucleic acid sequence data were used to construct a continuous sequence of each cPCR product. All sequencing reactions were carried out at the Purdue Genomics Core Facility (Purdue University, West Lafayette, IN, USA). The 16S rDNA sequences obtained were compared to GenBank® entries using the BLASTn tool provided by NCBI (http://www.ncbi.nlm.nih.gov/blast/Blast.cgi).

### Phylogenetic analysis

The 16S rRNA gene sequences from the rat isolates were aligned with sequences from GenBank® database using Clustal W2 (EMBL-EBI). Evolutionary analyses and phylogenetic tree were constructed using the software Mega 6.0 [31] with the neighbor-joining method [32] from a distance matrix corrected for nucleotide substitutions by the Kimura two-parameter model [33]. The data set was resampled 1,000 times to generate bootstrap values [34].

### Statistical analysis

A Chi-Square test was used to compare the gender, location and infection. A descriptive analysis of the variable (positive/negative for hemoplasma infection) collected from each group was performed. The Student’s *T* test was used for univariate analysis variables such as PCV, TPP, presence of structures on cells, and weight of the rats. The results were considered significantly different when *p* < 0.05. The statistical analysis for this paper was generated using SAS software (Copyright, SAS Institute Inc., Cary, NC, USA).

## Results

Among all rats, 30 (47.6 %) were female and 33 (52.4 %) were male. Among males 23/33 (69.6 %) were infected and 10/33 (30.4 %) showed no infection. Regarding the females, 17/30 (56.5 %) were infected, while 13/30 (43.5 %) showed no infection. Hemoplasma infection was not associated with the gender of the rats investigated (*p* = 0.11).

Although the average weight of non-infected rats (208.04 g ± 111.25) was lower than the weight of infected rats (256.12 g ± 124.00), no statistically significant difference (*p* = 0.15) was found when the groups were compared. As for place of origin, the number of infected rats was higher among those who were captured at the Passeio Público (16/23 = 69.6 %) and the Curitiba Zoo (15/20 = 75.0 %), compared with rats kept in the nursery of the Passeio Público (9/20 = 45.0 %). However, there was no statistically significant difference related to infection among the three groups (*p* = 0.11).

Quantitative PCR for hemoplasma infection using the SYBR® Green technique showed that 40 (63.5 %) out of the 63 samples tested positive. There was no statistically significant difference for weight, PCV and TPP between infected and non-infected groups.

The mean PCV of rats obtained from the nursery of Passeio Público (41.3 % ± 2.53) was greater than that of rats captured at the Passeio Público (37.4 % ± 3.76 and Zoo (34.7 % ± 3.66), but not enough to be statistically significant (*p* = 0.31). Similarly, the mean PCV of infected and non-infected rats when compared was slightly higher in the negative group (38.5 % ± 4.29) than in the positive group (37.4 ± 4.24). This difference was also not significantly different (*p* = 0.31).

In general, PCV values for infected and non-infected animals showed variable values for the different sites of origin/capture; infected animals of the Passeio Público had higher PCV (37.7 % ± 4.18) than non-infected animals (36.7 ± 2.81), while animals captured at the Zoo showed opposite results (non-infected group PCV: 35.6 % ± 5.50; infected group PCV: 34.5 % ± 3.04). The rats from the nursery of the Passeio Público showed more consistent and nearly equal PCV values in both the infected (41.7 % ± 1.41) and non-infected groups (41.0 % ± 3.22).

There were no significant associations between the total plasma protein mean of non-infected animals (6.33 g/dL ± 0.77) versus infected animals (6.69 g/dL ± 0.90) (*p* = 0.09) nor for the different groups or different places of origin.

### Microscopy

Some PCR positive samples had visible hemoplasma-like structures, whereas others had no visible structure on the red blood cells. Further, some negative PCR samples had structures such as Howell-Jolly bodies and/or stain precipitates, which are often misidentified as hemoplasmas. The mean number of hemoplasma–like structures within 1000 red blood cells was compared with infection status. In non-infected animals, the mean was 0.09 ± 0.08, whereas for infected animals was 0.09 ± 0.08. There was no statistically significant difference between the values of the two groups (*p* = 0.87).

### Molecular analyses

Samples testing positive by cPCR for the GAPDH gene showed a band of approximately 400 bp; none of the 63 samples failed to provide amplification and therefore no sample was excluded from the study. Of all samples, 40/63 (63.5 %) were positive for hemoplasma infection by SYBR® Green qPCR. Seventeen (42.5 %) of the 40 positive samples were initially considered suspect, having a C_T_ between 34 and 38 cycles; these samples were successfully reamplified using cPCR, confirming them as weak positives. Sixteen (69.5 %) out of 23 samples from the Passeio Público, were positive by the SYBR® Green technique: 10/16 (62.5 %) positive, and 6/16 (37.5 %) weak positive. Among samples from the Zoo, 15/20 (75.0 %) were positive: 10/15 (66.7 %) positive and 5/15 (33.3 %) weak positive. Nine out of 20 (45.0 %) samples were infected among samples from the nursery: 3/9 (33.3 %) positive, and 6/9 (66.7 %) weak positive. For purposes of statistical analysis and interpretation, the results were grouped into positives and negatives.

Positive samples by SYBR® Green were selected for sequencing according to their (melting temperature) T_m_. Twenty-six samples, representing at least 50 % of each T_m_ (72, 73, 74, 75 and 76) showed in the qPCR results, were amplified and sequenced using the primers designed to amplify a product of approximately 600 bp. Twenty-five out of the 26 samples showed identity (98- 100 %) with *M. haemomuris*, when compared to sequences deposited in GenBank® using BLASTn. One of the sequenced samples showed 99 % identity with an uncultured mycoplasma (ac. number KJ739311.1) and 94 % identity with the known feline hemotropic mycoplasma ‘*Candidatus* Mycoplasma turicensis’ (ac. number JQ689949.1). The highest percentages of identity and accession numbers of the sequences compared are shown in Table 2.

**Table 2:** Results of sequencing of selected SYBR**®** green positive samples for hemoplasma. Identities of partial sequences of the 16S rRNA gene (~600 bp) of hemoplasmas of 26 samples amplified, sequenced and compared to sequences deposited in GenBank**®**. Species identified, percentage of identity and accession numbers

Species	n	%	Accession number
*M. haemomuris*	12	100	AB758439.1
	4	99	
	2	100	AB820289.1
	5	99	
	1	98	
	1	100	AB918692.1
*Uncultured Mycoplasma sp. clone S_266 16S ribosomal RNA gene, partial sequence*	1	99	KJ739311.1
*‘Candidatus* M. turicensis’	1	94	JQ689949.1

Two samples were selected for further sequencing of the nearly entire 16S rRNA gene (~1300 bp) using newly designed primers; sample R37, whose sequence from the ~600 bp fragment was 100 % identical to *Mycoplasma haemomuris*, and sample R25 whose sequence of ~600 bp was 94 % identical to ‘*Candidatus* M. turicensis’. Both samples had clear dissociation curves (single peak) on SYBR® Green and clear sequence chromatograms. Sample R37 was once again 100 % identical to the 16S rRNA gene of *M. haemomuris* (AB758439.1), whereas sample R25 had 99 % identity to a sequence from a hemotropic mycoplasma identified in *Rattus norvegicus* in Hungary (ac. number KJ739311), and 98 % identity to a sequence from a hemoplasma found in a small rodent of the species *Micromys minutus* in this same country (ac. number KC863983.1). Further, this sample showed 95 % identity to the feline hemotropic mycoplasma ‘*Candidatus* M. turicensis’ (ac. number JQ689950.1). Identity between samples 37 and 25 was 91 %.

### Phylogenetic analysis

**Fig. 1:**
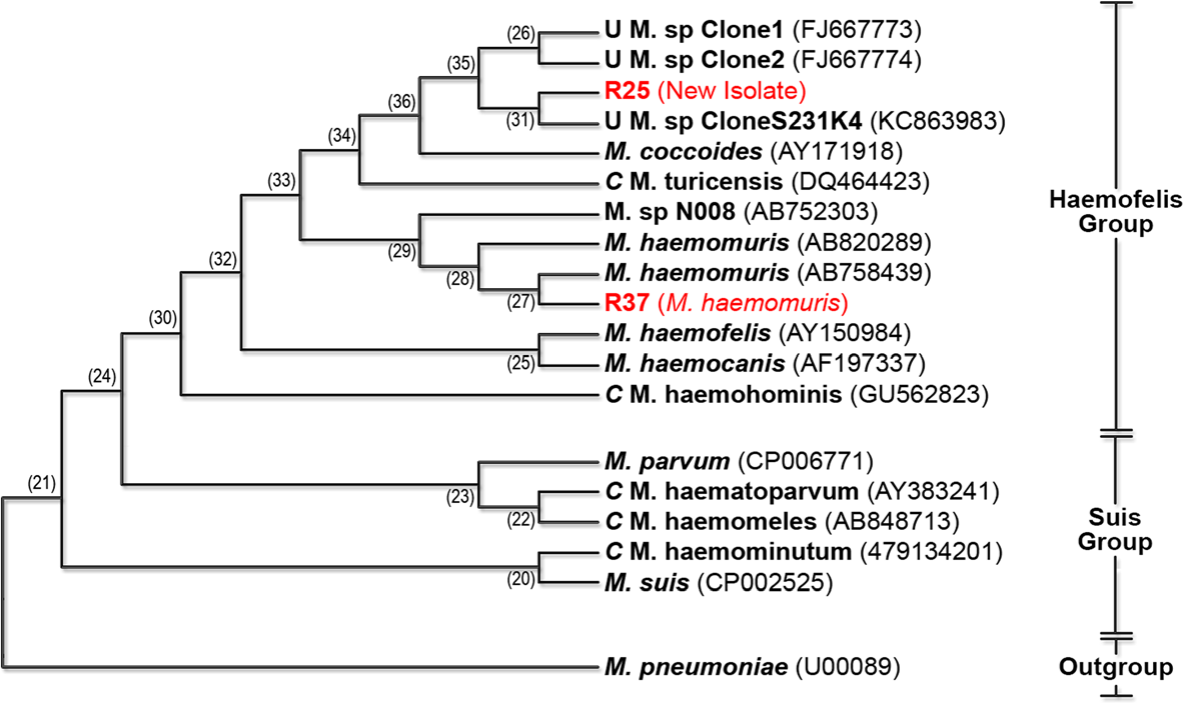
Phylogenetic tree based on 16S rRNA gene sequences, showing the relationship between the two isolates from rats (R25 and R37) and other hemotropic mycoplasmas. GenBank® accession numbers are included. The evolutionary history was inferred using the Neighbor-Joining method [32]. The optimal tree with the sum of branch length = 5,36444540 is shown. The percentage of replicate trees in which the associated taxa clustered together in the bootstrap test (1000 replicates) are shown next to the branches [34]. The evolutionary distances were computed using the Maximum Composite Likelihood method [45] and are in the units of the number of base substitutions per site. The analysis involved 19 nucleotide sequences. All positions containing gaps and missing data were eliminated. There were a total of 899 positions in the final dataset. Evolutionary analyses were conducted in MEGA6 [31]

Phylogenetic analysis based on 16S rRNA gene sequences amplified from sample R25 (1154 bp, ac. number KM203857) and R37 (1148 bp, ac. number KM258432) showed that both isolates cluster with the Haemofelis group (Fig. 1). This group includes ‘*Candidatus* M. turicensis’, *M. coccoides*, *M. haemomuris*, *M. haemobos*, *M. haemofelis* and *M. haemocanis*, as well as the novel isolate identified in *Micromys minutus*. Other closely related hemotropic mycoplasmas included a hemoplasma of capybaras (FJ667773 and FJ667774), in addition to *M. coccoides* (AY171918) and ‘*Candidatus* M. turicensis’ (DQ464423) reported in rodents and cats, respectively.

### Nucleotide sequence accession numbers

The nucleotide sequences of the novel hemoplasma and the *M. haemomuris* isolated from free-living rats were submitted to the GenBank® database under the accession numbers KM203857 (Sample R25 – New Isolate) and KM258432 (Sample R37 – *M. haemomuris*), respectively.

## Discussion

This is the first report of hemotropic mycoplasma infection of rats in Brazil and molecular characterization of the species involved. Our findings suggest that *M. haemomuris* infection is common in rat, however it is not associated with overt clinical disease. Rats in this study appear to be chronically infected, showing no differences in weight, PCV, and TPP when compared to non-infected rats. Although chronic hemoplasma infections may not cause clinical signs or hematologic alterations [13], there is always risk of spreading the organism to other susceptible animals. It is also recognized that when chronically infected animals are subjected to stress, coinfection, splenectomy, or immunosuppression, they may develop overt, systemic disease [3]. The presence of chronic hemoplasma infections is recognized as a common occurrence in several other animal species.

Despite the development of highly sensitive PCR assays, microscopic analysis of Romanowsky stained blood smears is still the method of choice for detecting hemotropic mycoplasmas in many laboratories [16]. The lack of correlation between microscopy and PCR results in this study agrees with previous reports describing the low sensitivity of the microscopic method [16]. Thus, microscopy greatly underestimates the extent of infection. This finding was anticipated as the bacteremia in chronically infected animals is often low and it is sometimes sporadic [35, 36]. However, using qPCR the presence of infection can be detected and subsequently verified by sequencing of the amplified products. Nearly all samples subjected to sequencing in this study were consistent with *M. haemomuris*, which is known to infect wild and laboratory rodents [35]. This study again emphasizes the need to use molecular methods as the ‘gold standard’ to determine whether or not an animal is infected, especially for detection of chronic hemoplasma infections.

As expected, we found a high percentage of hemoplasma-infected rats among the groups captured in the Passeio Público and Curitiba Zoo (31/43 = 72 %) when compared to laboratory raised rats. A similar percentage (80 %) was reported in free-ranging capybaras, a large rodent species native to South America [5], whereas Willi et al. [22] reported about half (53 %) of wild *Apodemus* mice in Switzerland infected with *M. coccoides*. The capybaras showed increased prevalence of infection among free-ranging (80 %) animals when compared to those in captivity (20 %). Similarly, a higher occurrence of hemoplasma infection in free-ranging wild cats versus those in captivity has been reported [22].

Surprisingly, the occurrence of *M. haemomuris* infection in rats from the nursery of the Passeio Público was also high and not statically different than that of free-ranging rats. These rats, used exclusively for feeding the carnivorous animals kept in the park, were sampled as a measure to access their health status as well as to serve as negative controls in the study herein. It was not possible to determine the source of infection in these animals; they were obtained from a commercial laboratory rat vendor and only briefly held in the nursery. The vendor does not routinely perform microscopic or molecular testing for detection of hemoplasma infection. Nevertheless, the presence of hemoplasma infection in the laboratory rats, suggests that hemoplasma infection is widespread in this animal species. It is particularly important to recognize that this potential confounding factor needs to be eliminated from colonies of rodents used in experimental studies [37]. Clinically silent infections are of major importance in research because they often go undetected until their presence is heralded by aberrations in experimental data. Hemoplasma infections are prime examples due to their extreme subtlety and profound influence on a great variety of studies [38–40].

The likelihood of a species other than *M. haemomuris* or another stain of *M. haemomuris* among the positive samples in this study suggests the presence of hemoplasma diversity in the population of infected free-ranging rodents in Brazil. The similarity between this second species with the feline hemoplasma ‘*Candidatus* M. turicensis’ and the phylogenetic distance from *M. haemomuris* suggests that this is likely a novel species. Moreover, the identity between the *M. haemomuris* isolate and this second hemoplasma in this study suggests that this is a new species rather than a different strain. Nevertheless, other analysis on a genomic basis should be conducted to further investigate this hypothesis. The 16S rRNA gene sequences have been widely used in microbiology to identify new species of uncultivable microorganisms and have also been the basis for reclassification of hemotropic mycoplasma species [2]. The phylogenetic analysis of the 16S rDNA gene of our rat isolates showed both of these bacteria belong to the Haemofelis cluster that includes ‘*Candidatus* M. turicensis’, *M. coccoides*, *M. haemomuris*, *M. haemobos*, *M. haemofelis* and *M. haemocanis*. It is important to determine which hemoplasma species and/or strains are involved in an infection as their pathogenicity may differ [41].

Some authors have proposed that rodents could represent reservoirs for feline hemoplasmas due to the close similarity between bacterial species [42], and that the relationship between ‘*Candidatus* M. turicensis’ and hemoplasmas that infect rodents suggest possible interspecies transmission of these agents [43, 44]. Interestingly, the sequence of the 16S rRNA gene of the novel species found in this study had 98 % identity to the sequence isolated from a hemoplasma infecting the blood of a harvest mouse (*Micromys minutus*), a small rodent Native Asian and Europe. It is possible that both isolates belong to the same not yet described species. However, a more complete phylogenetic analysis should be performed to better elucidate the relationship between these isolates. The nucleotide sequences of the 16S rRNA gene and the ITS region may further clarify the genetic relationship among the hemotropic pathogens isolated from mice, rats and hamsters [12]. Clinical and epidemiological relevance of this novel species remain uncertain.

The main limitation of this study was the inability to monitor infection, as the animals were evaluated at a single time point, and the use of PCV and TPP for detection of anemic animals rather than a complete blood count (CBC). A more extensive hematologic analysis could have provided additional information but was not undertaken in the current study. This decision was made in part because hemoplasmas are notorious for causing silent infection that may not appreciably alter hematologic variables, and also because comorbidities and infections in the studied population could not be controlled for, and may have interfered with meaningful interpretation of such variables. Future studies in experimentally infected rats may be needed to better assess the impact of infection on other hematologic parameters. Although the assessment of general health status of the captured rats was likely incomplete and based only on visual inspection, the animals appeared healthy, with the exception of a few variables, such as scars, superficial wounds, and ectoparasites.

Another limitation is the fact that the qPCR assay used in this study [30] does not provide absolute quantification. This protocol uses universal hemoplasma primers designed for screening of various hemoplasma species, and specific standard curves would be necessary for accurate quantification. Such standard curves were not available for this study.

## Conclusions

To the author’s knowledge, this is the first study conducted in Brazil using PCR technique to detect hemoplasmas in wild and laboratory-raised rats. The presence of *Mycoplasma haemomuris* was found in more than 63 % of the rats tested, including almost half of the laboratory-raised animals. These findings suggest that hemoplasmas are likely endemic in the region. The absence of statistically significant alterations in the hematologic parameters between infected and non-infected rats is consistent with the low pathogenicity and/or silent infection characteristics of these microorganisms. Further, our findings reiterates the need for investigation and control of these microorganisms in colonies of laboratory animals as well as the need for a better understanding of the effects of chronic infection in these animals. Lastly, the presence of a novel hemoplasma species infecting rats suggests the possibility that there are additional strains or possibly other species of hemotropic mycoplasmas yet to be described. Additional studies are also needed to understand the clinical and epidemiological impact of chronic hemoplasma infections on local fauna and wild animals kept in the parks and to clarify their role as possible zoonotic agents.

### Availability of supporting data

The nucleotide sequencing data supporting the results of this article are available in the GenBank® repository, http://www.ncbi.nlm.nih.gov/nuccore/KM203857 and http://www.ncbi.nlm.nih.gov/nuccore/KM258432.
